# Near ideal synaptic functionalities in Li ion synaptic transistor using Li_3_PO_x_Se_x_ electrolyte with high ionic conductivity

**DOI:** 10.1038/s41598-019-55310-8

**Published:** 2019-12-11

**Authors:** Revannath Dnyandeo Nikam, Myonghoon Kwak, Jongwon Lee, Krishn Gopal Rajput, Writam Banerjee, Hyunsang Hwang

**Affiliations:** 1 Center for Single Atom-based Semiconductor Device, Pohang University of Science and Technology (POSTECH), Pohang, 790-784 Republic of Korea; 2 Department of Material Science and Engineering, Pohang University of Science and Technology (POSTECH), Pohang, 790-784 Republic of Korea

**Keywords:** Electronic devices, Electronic devices

## Abstract

All solid-state lithium-ion transistors are considered as promising synaptic devices for building artificial neural networks for neuromorphic computing. However, the slow ionic conduction in existing electrolytes hinders the performance of lithium-ion-based synaptic transistors. In this study, we systematically explore the influence of ionic conductivity of electrolytes on the synaptic performance of ionic transistors. Isovalent chalcogenide substitution such as Se in Li_3_PO_4_ significantly reduces the activation energy for Li ion migration from 0.35 to 0.253 eV, leading to a fast ionic conduction. This high ionic conductivity allows linear conductance switching in the LiCoO_2_ channel with several discrete nonvolatile states and good retention for both potentiation and depression steps. Consequently, optimized devices demonstrate the smallest nonlinearity ratio of 0.12 and high on/off ratio of 19. However, Li_3_PO_4_ electrolyte (with lower ionic conductivity) shows asymmetric and nonlinear weight-update characteristics. Our findings show that the facilitation of Li ionic conduction in solid-state electrolyte suggests potential application in artificial synapse device development.

## Introduction

The recent advances in development of redox transistor such as lithium-ion synaptic transistor (LIST) to build an artificial neural networks exhibit tremendous advantages over the existing memristors technology^1–3^. In a LIST device, the read and write operations are performed separately using a “gate electrolyte” to switch the channel conductance by injecting or extracting Li^+^ ion from solid electrolyte or channel^1,2,4^. The inserted or extracted ion from the channel changes the doping state through which multilevel conductance level can be achieved. However, the low ionic conductivity of currently available electrolytes creates nonlinear switching and retention issues in LIST devices. The ionic liquid based electrolytes shows better prospects to achieve some synaptic functionalities^5,6^. However, the use of traditional liquid electrolyte limits the practical application of synaptic devices, and it forms lithium dendrites that affect its long-term stability^4^. The use of solid-state electrolyte offers several advantages over liquid electrolytes such as better stability and low energy operation by reducing the switching energy. Recently, the polymer-based solid-state electrolyte, polyethylene oxide: lithium perchlorate (PEO: LiClO_4_), has been proven to be capable of achieving excellent synaptic functionality owing to the high ionic conductivity of PEO substitute^2^. However, the polymer-based solid-state electrolyte PEO: LiClO_4_ is soluble in a commonly used lithographic solvent that complicates the creation of the top gate architecture. Therefore, these devices are always fabricated with a lateral gate architecture with gaps of several micrometers from a channel that induces a large electric double layer formation, which causes an adverse effect on ion migration reversibility. The lithium phosphate (Li_3_PO_4_) is the most well-known and studied lithium oxide solid electrolytes^7^. The ionic conductivity of the pure Li_3_PO_4_ in bulk is relatively low at 10^−7^ S·cm^−1^ and in thin film 10^−8^ S·cm^−1 ^^8^. The nitrogen doping Li3PO4 was first reported by Bates *et al*. Experimental evidences confirm that nitrogen replaces the bridging oxygen (P-O-P) and nonbridging oxygen (P-O) in Li_3_PO_4_ and forms a cross link structure.^9^. Bates *et al*. suggested that cross linked structure between P and N atoms result a high ionic conductivity of up to 10^−6^ S·cm^−1^ at 25 °C in micrometer scale thick LiPON film, it is sufficient to achieve high performance in large scale energy storage devices. It is worth to noting that scaling down the LIPON thickness to nm scale greatly reduces the ionic conductivity between 10^−10^ to 10^−7^ S·cm^−1^ that hinders the applicability of LiPON in nanoscale electronic devices such ionic transistor^10^. Therefore, due to above mentioned LiPON limitation, alternative thin film electrolyte material with at least 10^−6^ S·cm^−1^ ionic conductivity is highly desirable for nanoscale synaptic transistor. Apart from the nitridation (nitrogen doping) in Li_3_PO_4_, another scheme to increases the ionic conductivity in thin film is the isovalent substitution of oxygen (both bridging and apical oxygen) by anion such as S and Se but those result not yet verified experimentally^11^. The theoretical study confirms that (S or Se) bridge with P that allow the fast Li^+^ ion conduction because the S or Se forms the weak ionic bond with Li ion (Li–Se or Li-S)^12^. It is still not fully understood the underlying mechanism behind this, but it is empirically proven without any doubt. In spite the recent advances in developing high Li^+^ conducting electrolyte, in this study we have demonstrate Se doping in Li_3_PO_4_ electrolyte and develop a LIST device compatible Li_3_PO_x_Se_x_ solid electrolyte.

To the best of our knowledge, no study has clarified the influence of ionic conductivity of electrolyte on the synaptic property of LIST devices. Without a way to address the influence of ionic conductivity and develop high ionic conductive in thin film electrolyte, the LIST cannot function as an ideal synaptic device. Therefore, to enhance the practicality of employing LIST as an ideal synapse device, we devoted our study to develop a LIST compatible thin film electrolyte and systematically explore its effect on synaptic functionalities.

## Result and Discussion

**Figure 1:**
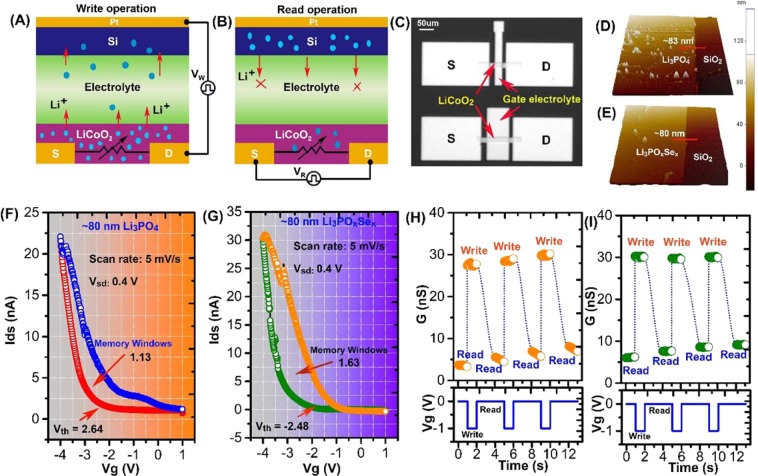
Li^+^ ion-based three-terminal redox transistors and their characteristics. (**A**) Schematic representation of “write” operation. A single redox transistor unit consists of Li^+^ ion rich channel and ionically conducting electrolyte. Selective programming occurs when V_W_ is greater than the OCP of redox transistor. During Li^+^ ion extraction (injection) from/to LiCoO_2_, its conductivity increases or decreases. **(B)** Schematic representation of “read” operation. Below the programming condition (V_W_ < OCP), the extraction or injection of Li^+^ ion stops and retains the nonvolatile state. This is the readout state for the redox transistor, performed by applying a small V_R_ between the source-drain terminal. **(C)** Optical image of the fabricated synaptic transistor. The LiCoO_2_ channel fabricated between source (S) and drain (**D**) terminal. **(D,E)** 3D topographic AFM height images of Li_3_PO_4_ and Li_3_PO_x_Se_x_ electrolytes on SiO_2_ substrate. **(F,G)** The transfer characteristics with V_g_ weeping rate of 4 mV/s of a redox transistor comprising Li_3_PO_4_ and Li_3_PO_x_Se_x_ electrolytes. The hysteresis behavior in channel current during forward and reverse scans represents the nonvolatility of redox transistor. In comparison to Li_3_PO_x_Se_x_, the Li_3_PO_4_-based redox transistor shows a small hysteresis leading to small memory windows (1.13 V) with high device operation voltage (−2.64 V). (**H,I**) Postsynaptic current response to presynaptic spike in synaptic transistors consisting of Li_3_PO_4_ and Li_3_PO_x_Se_x_ electrolytes respectively. The stable or unstable channel conductance states during read operation generated by a sequence of presynaptic gate spikes representing the volatility or nonvolatility.

The synaptic device used in this study had a three-terminal configuration consisting of lithium cobalt oxide (LiCoO_2_) as a channel, and ionic conductive material such as lithium phosphate (Li_3_PO_4_) or Se doped lithium phosphate (Li_3_PO_x_Se_x_) as a solid state gate electrolyte. Figure 1(A,B) represents the simultaneous write-read operations. During the write operation, the program voltage pulse is applied to the gate electrolyte through the silicon (Si) reservoir. The voltage pulse applied to the gate terminal creates a local ionic gating effect, which extracts/injects Li^+^ ion from/to the LiCoO_2_ channel layer. The LiCoO_2_ is layered structure in which Li situated between layers of octahedral unit of CoO_6_ andmetallic to insulator phase transition occurred in LiCoO_2_ as a result of extraction of fraction of Li ion^13^. In ionic transistor, conductance switching in LiCoO_2_ is governed by the change in Li ions concentration^1^. **(**Fig. 1A**)**. Consequently, to read the channel conductance change during the write operation, a small DC bias is applied between the source-drain terminal **(**Fig. 1B**)**. Optical microscopic image of the fabricated LIST device on a Si/SiO_2_ substrate is shown in **(**Fig. 1C**)**. We fabricated sets of LIST devices with various LiCoO_2_ channel length. As depicted in **(**Fig. 1C**)**, the channel dimensions in upper and lower devices are W/L ~20 µm/50 µm and ~20 µm/100 µm respectively. The thickness and surface roughness of electrolyte plays a cricial role in ionic transistor. The surface morphology and thickness of as deposited electrolytes was evaluated using Atomic Force Microscopy (AFM). Figure 1(D,E) shows the typical 3D topographic AFM images of Li_3_PO_4_ and Li_3_PO_x_Se_x_ electrolytes on SiO_2_ substrate. It was discovered that the deposited Li_3_PO_4_ electrolyte shows ~83 nm smooth height surface with small pinnacles in its 3D topographic scan **(**Fig. 1(D)**)**. The surface topography of Li_3_PO_x_Se_x_ electrolyte shows the smooth surface with ~80 nm thickness as shown in Fig. 1(E).

Controlling the ionic conduction in solid state devices is the crucial step and we believe that to trigger all synaptic functionalities using the LIST device, choosing a proper electrolyte is an important step. No other studies have yet reported the investigation of the effect of ionic conductivity of electrolytes on synaptic functionalities. To support our statement, we fabricate two redox transistors consisting of two different electrolytes with varying ionic conductivity. In this study, a low ionic conducting electrolyte (Li_3_PO_4_) and a high ionic conducting electrolyte (Li_3_PO_x_Se_x_) were used to explore the effect of ionic conductivity on synaptic functionalities. The switching behavior of the channel and its nonvolatility in LIST devices depend on the movement of Li^+^ ions between LiCoO_2_ and Si, which is directly related to the ionic conductivity of the electrolyte. The transfer characteristics and the hysteresis loop behavior of the redox transistor can provide clear evidence on the influence of ionic conductivity of electrolyte. The transfer characteristics of as-fabricated redox transistors are measured by applying the varying gate bias at a swept rate of 5 mV s^−1^ and a source-drain voltage of 0.4 V. In both devices, the channel current increases as the gate voltage is swept to negative values, while the channel current decreases when the gate voltage is swept to positive values, indicating the reversible switching of channel from high resistance (HRS) state to low resistance state (LRS). Figure 1F shows the transfer characteristics of the LIST device consisting of Li_3_PO_4_ electrolyte. In the case of Li_3_PO_4_ electrolyte, the transfer curve clearly indicates that the hysteresis between the positive and negative swept is minimal owing to the low ionic conduction of Li^+^ ions through the electrolyte. Additionally, the Li_3_PO_4_ consisted LIST device shows the small memory windows of 1.13 V and high device operating potential of −2.64 V. The transfer characteristics of the LIST device consisting of Li_3_PO_x_Se_x_ electrolyte are shown in Fig. 1G. The large hysteresis loop in the transfer curve is attributed by the higher Li^+^ ion transport through the Li_3_PO_x_Se_x_ electrolyte layer. The resultant device shows large memory windows of 1.63 V with a lower operating potential of −2.48 V. The large hysteresis in Li_3_PO_x_Se_x_ consisted LIST device associated with nonvolatile conductance change in LiCoO_2_ channel. The magnitude of ion induced hysteresis in LIST devices is directly link to the net ionic conduction through electrolyte layer and can be further explained qualitatively by measuring the volatile/nonvolatile memory behavior. To study the nonvolatile behavior in LIST devices a series of presynaptic spikes (−1 V, 1 s) with a pulse interval of 1 s were applied to via electrolyte gate terminal and postsynaptic current change in channel were measured for 1 s as shown in Fig. 1(H,I)**)**. The presynaptic spike denoted as write operation, whereas postsynaptic spike denoted as a read operation. In case of Li_3_PO_4_ consisted LIST device each presynaptic spike raises the channel conductance to certain value (denoted as read) as shown in Fig. 1(H). Due the low ion conduction through Li_3_PO_4_ electrolyte the Li ion just accumulated at channel-electrolyte interface and gradually diffuse back to channel, resulting in massive decay in channel conductance. The decay in channel conductance during read operation represent the volatile memory behavior that causes the shallow hysteresis in Li_3_PO_4_ consisted LIST deices as shown in Fig. 1(F). On the other hand, the Li_3_PO_x_Se_x_ consisted LIST device each presynaptic spike induces very steady change in channel conductance (denoted as read) as shown in Fig. 1(I). The fast ionic conduction through Li_3_PO_x_Se_x_ electrolyte the Li ions no longer accumulate at the channel electrolyte interface, resulting in steady state channel conductance change. The steady channel conductance change in Li_3_PO_x_Se_x_ consisted LIST device depicted the nonvolatile memory behavior that causes the large hysteresis as shown in Fig. 1(G). The shallow hysteresis due to volatile conductance state or large hysteresis due to nonvolatile conductance state in LIST devices are governed by net moment of Li ion from channel to reservoir via electrolyte layer. To underpin our hypothesis, we have performed the theoretical simulation to calculate the real time Li^+^ ion concentration. More detailed description on the time dependent Li^+^ distribution simulation can be found in Supplementary Information (Section 1). To perform the simulation, we first obtained a real time discharge curve for both Li_3_PO_4_ and Li_3_PO_x_Se_x_ devices as shown in Fig. S1. Subsequently, we process the simulation by considering known device parameter such as thickness, ionic conductivity, area. Then we tune the various unknown parameter such as reaction rate, diffusion coefficient until a good fit is achieved for experimental data. Finally, we perform the simulation in real time mode to obtain the unknown concentration of Li^+^ ion in LiCoO_2_ layer. The parameter used in simulation are listed in Table S1. It is worth noting that there is a significant difference in Li^+^ ion concentration distribution in LiCoO_2_ layer was observed between Li_3_PO_4_ and Li_3_PO_x_Se_x_ consisted LIST devices (Fig. S2). The high Li^+^ ion concentration in in LiCoO_2_ layer is clear evidence of a fast back diffusion of Li ion due to low ionic conduction in Li_3_PO_4_ consisted device.

In order to underpin the ionic conduction phenomenon in Li_3_PO_4_ and Li_3_PO_x_Se_x_ electrolytes, we extend our study to understand their bonding structure. Numerous studies predicted the influence of bonding structure in Li_3_PO_4_ on Li ion mobility^14^. The Li_3_PO_4_ comprised of PO_4_^3−^ tetrahedral unit, each of which is bonded to other tetrahedral through bridging oxygen (P–O–P)^15^. Two outer oxygen atoms in PO_4_^3−^ unit have ionic bond with two Li atom denoted as a non-bridging oxygen (Li^+^… O–P). The remaining one oxygen atom in PO_4_^3−^ unit form the π̀ bond with central phosphorous atom denoted as a non-bridging oxygen (O=P)^16^. The Li atoms have the freedom to migrate to the empty π bonded oxygen atom site (and the π bond moves to the vacated Li site), which constitutes the most basic form of ion transport. Due to the high electronegativity of oxygen atom forms the strong electrostatic interaction with Li atom (Li^+^… O–P) as a result lack of intrinsic mobile Li vacancies in Li_3_PO_4_ and thereby decreases its ionic conductivity^14,17^. More specifically, changing the bonding structure by substituting non-bridging oxygen (Li^+^… O–P) and bridging oxygen (P–O–P) by nitrogen or isovalent anion would result in a reduction of the overall electrostatic interaction with Li^+^. This weak electrostatic interaction destabilizes the Li^+^, result in formation of extrinsic Li vacancies in doped Li_3_PO_4_ and thereby increases its ionic conductivity^11,18^. Additionally, iosovalent anion substitution of in Li_3_PO_4_ causes disordered Li-ion sublattice that provide more stable Li interstitial sites thereby contribute high ionic conductivity^19,20^.

**Figure 2:**
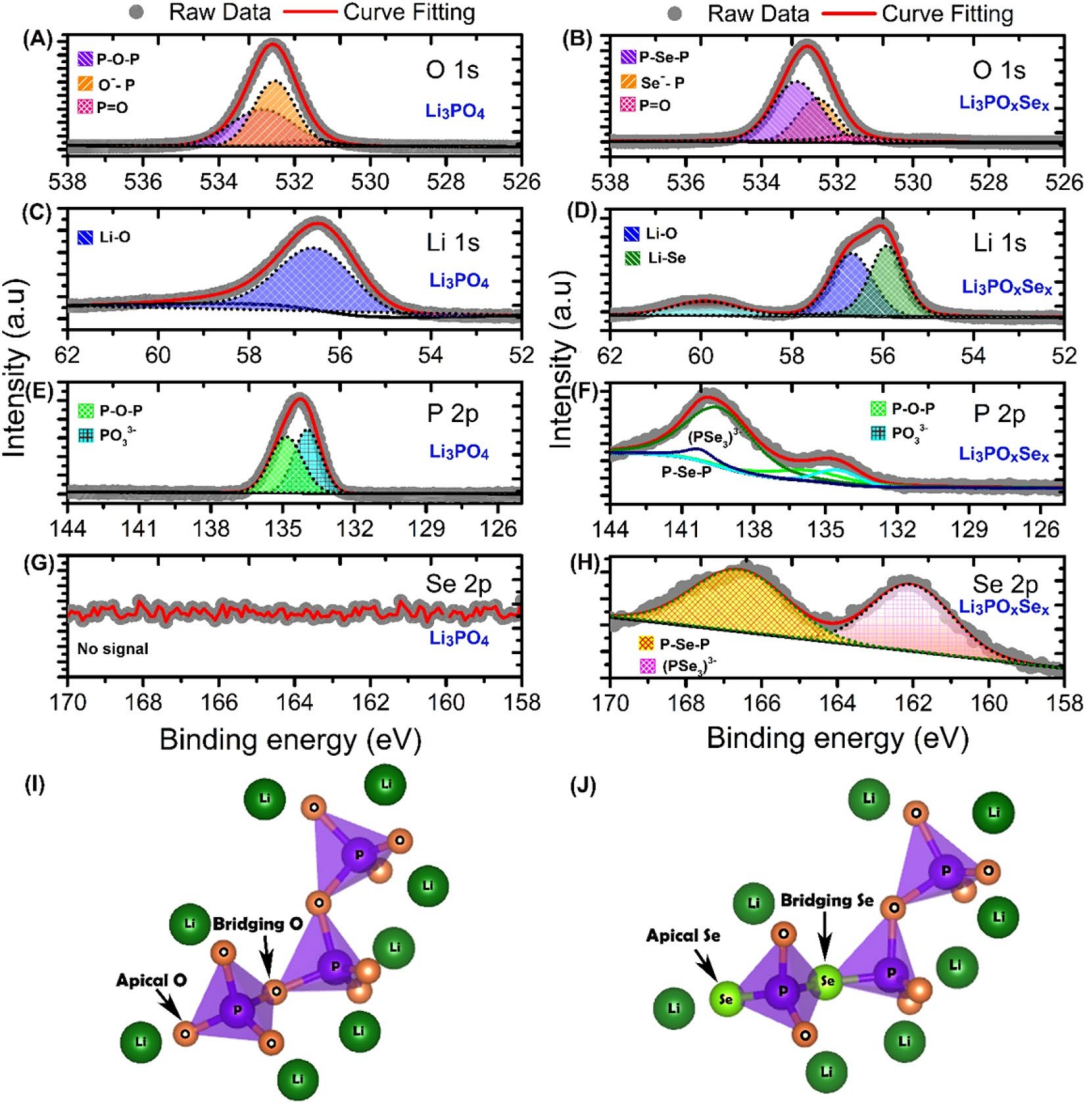
Chemical structural analysis of as fabricated Li_3_PO_4_ and Li_3_PO_x_Se_x_ electrolytes using high resolution core level XPS (**A)** O 1 *s* core spectra in Li_3_PO_4_ **(B)** O 1*s* core spectra in Li_3_PO_x_Se_x_ **(C)** Li 1*s* core spectra in Li_3_PO_4_ **(D)** Li 1*s* core spectra in Li_3_PO_x_Se_x_ **(E)** P 2*p* core spectra in Li_3_PO_4_ **(F)** P 2*p* core spectra in Li_3_PO_x_Se_x_ **(G)** Se 2*p* core spectra in Li_3_PO_4_ **(H)** Se 2*p* core spectra in Li_3_PO_x_Se_x_ **(I)** Model chemical structutre of Li_3_PO_4_ electrolyte and **(J)** Model chemical structutre of Li_3_PO_x_Se_x_ electrolyte.

**Figure 3:**
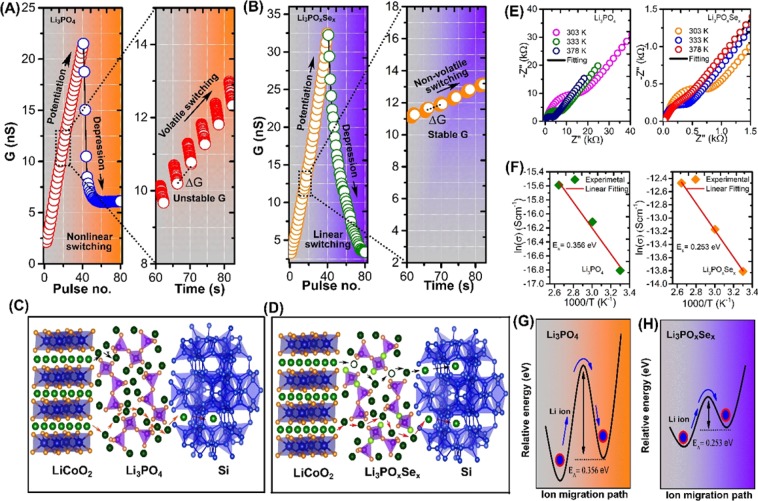
Interionic switching mechanism and channel conductance change stimulated by programmed pulses in redox transistor comprising different electrolytes. The redox transistors were operated by 40 programmed down/up gate voltage pulses (±1 V, 2 s with space 2 s). **(A)** Asymmetric channel conductance change in Li_3_PO_4_-based redox transistor associated with accumulation Li^+^ ion at channel-electrolyte interface due to lower ionic condcution in Li_3_PO_4_. Zoom-in view shows a massive and unstable conductance change per pulse (∆G). **(B)** Symmetric stable channel conductance switching occurred in Li_3_PO_x_Se_x_-based redox transistor due to the uniform Li^+^ ion migration associated with its high ionic conductivity. The zoom-in view shows the discrete and stable conductance state. **(C)** Schematic of interionic switching dynamics for Li_3_PO_4_ consisted LIST device. The low ionic conduction electrolyte associated with the absence of Li vacancy site in Li_3_PO_4_ that avoids the migration of Li^+^ ion from LiCoO_2_ to Si reservior. Consequently, Li^+^ gets accumulated at channel-electrolyte interface. **(D)** Schematic of interionic switching dynamics for Li_3_PO_x_Se_x_-consisted LIST device. The fast ionic conduction associated with presence of extrinsic Li ion vacancies and interstitial configurations in Li_3_PO_x_Se_x_, that allow the uniform Li^+^ ion migration between LiCoO_2_ to Si reservior. **(E)** Temperature dependent EIS measurements for Li_3_PO_4_ and Li_3_PO_x_Se_x_ electrolytes, respectively. EIS measurement was performed at a test frequency region varying from 0.1 Hz to 1 MHz, using two terminal cells consisting of a sandwiched electrolyte layer between the top and bottom Pt electrodes. The dots are the measured data and the lines are the fitted data using the equivalent circuit model. **(F)** Arrhenius plots of the ionic conductivity of the Li_3_PO_4_ and Li_3_PO_x_Se_x_ electrolytes **(G)** Activation energy for Li^+^ ion migration in Li_3_PO_4_ electrolyte. (**H**) Activation energy for Li^+^ ion migration in Li_3_PO_x_Se_x_ electrolyte.

In order to further confirm the chemical composition and nature of the chemical bonding in Li_3_PO_4_ and Li_3_PO_x_Se_x_ electrolytes, high resolution X-ray photoelectron spectroscopy (XPS) was performed and are shown in Fig. 2. Prior to the XPS analysis, both Li_3_PO_4_ and Li_3_PO_x_Se_x_ samples were etched up to 10 nm to avoid the effect of surface oxidation. The initial XPS survey scan confirmed the presence of Li, P, O, Se and C in Li_3_PO_4_ and Li_3_PO_x_Se_x_ samples respectively **(**Figs. S3 and S4**)**. The XPS core peak spectra of O 1 *s* for Li_3_PO_4_ electrolyte is shown in Fig. 2A. The O 1 *s* spectra deconvoluted into the three distinct peaks attributed respectively to non-bridging oxygen (P=O) at 531.5 eV, non-bridging oxygen (Li^+^… O–P) at 532.5 eV, and bridging oxygen (P–O–P) at 532.8 eV. The obtained fitted by Gaussian–Lorentzian XPS curves for O 1 *s* region are consistent with previously reported results on Li_3_PO_4_ electrolyte^21^. The non-bridging oxygen (Li^+^… O–P) and bridging oxygen (P–O–P) are the chemical reactive site toward elemental doping such as Se. The Li_3_PO_x_Se_x_ electrolyte was obtained by selenization of Li_3_PO_4_ due to partial replacement of oxygen species in Li^+^… O–P and P–O–P bonds by Se. The XPS core peak spectra of O 1 *s* for Li_3_PO_x_Se_x_ electrolyte is depicted in Fig. 2B. In Li_3_PO_x_Se_x_ electrolyte the peak of non-bridging oxygen at 531.5 eV is remain unchanged, clearly indicating (P=O) conserved during selenization of Li_3_PO_4_. During selenization, we notice the major changes in non-bridging oxygen (Li^+^… O–P) and bridging oxygen (P–O–P) sites. The selenization process greatly reduces the intensity second peak at 532.5 eV that was associated with non-bridging (Li^+^… O–P) chemical environment. This indicates a substitution of an oxygen atom by a selenium atom at non-bridging site to form (Li^+^… Se–P) bonds. The third peak located at 533.05 eV is associated with bridging (P-Se-P) chemical environment. The up shift of this peak during selenization is believed to be due the replacement of P-O-P bond by P-Se-P bond. The XPS core spectra of Li 1 s for Li_3_PO_4_ electrolyte is shown in Fig. 2C. The peak position of Li 1 *s* located at 56.59 eV is due the highly polarized bonding between Li and O (Li-O) and it indicating single form of chemical environment of Li at the non-bridging site (Li^+^… O–P). The spectra of Li 1 *s* for Li_3_PO_x_Se_x_ electrolyte is depicted in Fig. 2D and decomposed into two distinct peaks. The reduced intensity of 56.59 eV peak clearly indicating reduction of non-bridging oxygen site (Li^+^… O–P). The newly generated downshifted peak at 55.87 eV due to the formation of low polarized non-bridging selenium site (Li^+^… Se–P). Furthermore, to know the chemical environment of P in Li_3_PO_4_ electrolyte the P 2*p* slow scan peak were deconvoluted into a two set of peaks by considering the spin orbital splitted doublet P 2*p*_*1/2*_ and P 2*p*_*3/2*_ as shown in Fig. 2E. The first peak observed at 133.94 eV is the is the characteristics of a PO_4_^3−^ tetrahedral chemical environment^22^. Note that the peak at 133.94 is the combination of doublets 2*p*_*1/2*_ and 2*p*_*3/2*_. The peak observed at 134.83 eV reveals the existence of bridging P-O-P bond surrounded by a lithium chemical environment. To further confirm the chemical state of P in Li_3_PO_x_Se_x_ electrolyte, we extend our XPS analysis into P 2*p* regions as shown in Fig. 2F**)**. The XPS core spectra of Li_3_PO_x_Se_x_ electrolyte in P 2*p* region clearly shows that the peak intensity of P 2*p*_*1/2*_ (133.94 eV) and P 2*p*_*3/2*_ (134.84 eV) are reduced due appearance of new peaks at 139.57 eV, 140.26 eV due to formation of (PSe_3_^3−^) and P-Se-P chemical species. The XPS scan in the Se 2*p* region **(**Fig. 2G**)** shows no signal that clearly indicating the absence of Se in Li_3_PO_4_ electrolyte. We believe that the selenization process allow to replace the highly polarized bridging oxygen (P–O–P) and non-bridging oxygen (Li^+^… O–P) sites Li_3_PO_4_ electrolyte. Interestingly, we found the remarkable change in Se 2*p* region of Li_3_PO_x_Se_x_ electrolyte **(**Fig. 3H**)**. The two distinct peaks at 162.06 eV, 166.56 eV are associated with (PSe_3_^3−^) and P-Se-P chemical environment. The fractional elemental percentage in Li_3_PO_4_ and Li_3_PO_x_Se_x_ electrolytes obtained from XPS are listed in Tables S2 and S3. Finally, based on our XPS analysis result, we propose the model structures for Li_3_PO_4_ and Li_3_PO_x_Se_x_ electrolytes as shown in Fig. 2(I,J). In case of Li_3_PO_4_, the PO_3_^3−^ link to another PO_3_^3−^ thorough bridging oxygen (P–O–P) and three oxygen on each tetrahedral denoted as apical oxygen or non-bridging oxygen site (Li^+^… O–P). In Li_3_PO_x_Se_x_ the substitution of bridging oxygen (P–O–P) by Se stabilizes the Li vacancy by forming bridging selenium (P–Se–P) site. Also, the selenization Se substitution for a tetrahedral O in PO_3_^3−^ stabilizes a Li inferential ion by forming apical selenium or non-bridging selenium site (Li^+^… O–P).

To demonstrate the effect of ionic conductivity of electrolytes on the stability of finely spaced conductance state and their symmetry, LIST device was operated by sending several programmed voltage pulses to the gate electrolyte. The “write” operation consists of 40 down pulses (−1 V, 2 s with space 2 s) and 40 up pulses (+1 V, 2 s with space 2 s). The “read” operation is performed at 1 s right after each successive voltage pulse by applying a small 0.5 V DC bias to the source-drain terminal. As shown in Fig. 3A, when the negative voltage pulse (potentiation) is applied to the gate electrolyte, an Li^+^ ion starts to move from the LiCoO_2_ channel to the Si reservoir through the Li_3_PO_4_ electrolyte. The extracted Li^+^ ion from the channel changes the doping state in LiCoO_2,_ and the channel conductance gradually switches off up to certain pulses. Subsequently. a further increase in the negative voltage pulse on the gate electrolyte results in a sudden increase in channel conductance. The zoom in view shows that the conductance change per pulse (∆G) is more massive and unstable indicating high volatility. Similarly, when a series of identical positive pulses (depression) is applied to the same device, a sudden decrease in channel conductance is observed up to a certain pulse, and the subsequent channel conductance becomes saturated. This behavior of abruptness and saturation in channel conductance is due to the accumulation of Li ion at channel electrolyte interface, which is governed by low ionic conduction in Li_3_PO_4_ electrolyte. In contrast, Li_3_PO_x_Se_x-_based LIST device exhibit symmetric analog conductance change (Fig. 3B). The zoom in view shows the stable and nonvolatile conductance change. The high nonvolatility in Li_3_PO_x_Se_x-_based LIST device is governed by the high ionic conduction through electrolyte layer. As a result of fast ionic conduction through Li_3_PO_x_Se_x_ electrolyte the Li ions no longer accumulate at the channel electrolyte interface, resulting in analog channel conductance change. Figure 3(C,D) show the schematic of the interionic switching mechanism of Li_3_PO_4_ and Li_3_PO_x_Se_x_ electrolytes, respectively. As shown in Fig. 3C, Li_3_PO_4_ electrolyte based LIST device exhibit least Li^+^ ion migration from LiCoO_2_ channel to Si reservoir. The nonlinear switching behavior in Li_3_PO_4_ consisted LIST devices arises due to the low Li ion migration from LiCoO_2_ channel to Si reservoir through Li_3_PO_4_ electrolyte. The overall electrochemical reactions involving Li ions migration in LiCoO_2_/Li_3_PO_4_ /Si are as follows:

1 $${{\rm{LiCoO}}}_{2}\mbox{--}(1-{{\rm{x}}}_{1}){{\rm{e}}}^{-}\leftrightarrow {{\rm{Li}}}_{{\rm{x}}1}{{\rm{CoO}}}_{2}+(1-{{\rm{x}}}_{1}){{\rm{Li}}}^{+}$$

2 $${{\rm{Li}}}_{3}{{\rm{PO}}}_{4}+{{\rm{x}}}_{2}{{\rm{Li}}}^{+}+{{\rm{x}}}_{2}{{\rm{e}}}^{-}\leftrightarrow {{\rm{Li}}}_{{\rm{x}}2}{{\rm{Li}}}_{3}{{\rm{PO}}}_{4}$$

3 $${\rm{Si}}+{{\rm{x}}}_{3}{{\rm{Li}}}^{+}+{{\rm{x}}}_{3}{{\rm{e}}}^{-}\leftrightarrow {{\rm{Li}}}_{{\rm{x}}3}{\rm{Si}}$$

The electrochemical reaction (1) and (3) are happened in channel and reservoir layer. The electrochemical reaction (2) is related to transport of Li^+^ ions through Li_3_PO_4_ electrolyte and it is most important step in LIST device because it determines how fast Li^+^ ions are transported toward Si reservoir layer and ultimately it determine the switching behavior of channel. It is well known fact that relatively low ionic conductivity of Li_3_PO_4_ thin film inherit it applicability in nano-batteries and nanoscale electronic devices. Previous experimental evidences predicted that the low ionic conductive electrolyte result in a Li accumulation at cathode/electrolyte interface. Those accumulated Li directly affect the reversible intercalation in LiCoO_2_. In Li_3_PO_4_ consisted LIST device the nonlinear switching behavior associated with accumulation of Li ion at channel/electrolyte interface. During potentiation the Li ion start to extract from channel LiCoO_2_ channel as a result conductance gradually increases. Due to low conduction through Li_3_PO_4_ extracted Li ion accumulated at the channel/electrolyte interface (LiCoO_2_/Li_3_PO_4_). Those accumulated Li ions can easily diffuse back to channel, which can be accounted for the high volatility as shown in zoom in view of Fig. 3A. During the depression, the opposite gate polarity all accumulated Li quickly diffuses back to channel, which can be accounted for abrupt conductance decay (Fig. 3A)). In Li_3_PO_4_ there are no intrinsic mobile Li vacancies so that the activation energy for Li ion migration is determined by the combination of the “formation” energy (E_f_) needed to produce a mobile Li ion by generating vacancy/interstitial site and migration energy E_m_. The theoretical prediction indicates that the lower ionic conductivity in Li_3_PO_4_ is associated with the large activation energy for Li ion migration. The larger E_A_ in Li_3_PO_4_ is due to the formation process of intrinsic mobile ions and that the interstitial ionic conduction mechanism is mostly responsible for ionic transport^23^. One way to avoid the formation energy bottleneck is to dope the Li_3_PO_4_ and generate extrinsic mobile Li ions so that the activation is determined by the migration energy alone i.e E_A_ = E_m_^24^. As well as elemental doping can cause disordered Li-ion sublattice that provide more Li interstitial site for easy Li ion migration^24^. To improve the synaptic functionalities, we replace the conventional Li_3_PO_4_ electrolytes with Li_3_PO_x_Se_x_. The interionic switching mechanism in Li_3_PO_x_Se_x_ consisted LIST device depicted in Fig. 3(D). The linear switching in Li_3_PO_x_Se_x_ consisted LIST device is associated with fast Li ionic conduction from LiCoO_2_ channel to Si reservoir through Li_3_PO_x_Se_x_ electrolyte. The XPS analysis confirm that selenization process allow the substitution of bridging oxygen (P–O–P) by Se that stabilizes the Li vacancy by forming bridging selenium (P–Se–P) site. Also, the selenization Se substitution for a tetrahedral O in PO_3_^3−^ stabilizes a Li inferential ion by forming apical selenium or non-bridging selenium site (Li^+^… O–P). The stable Li ion vacancy and interstitial allow the fast Li ion motion that result in high ionic conductivity in Se doped Li_3_PO_4_.

To further confirm the effect of Se-substitution on the ionic conductivity and activation energy of electrolytes, a temperature dependent electrochemical impedance spectroscopy (EIS) tests were performed at a test frequency region varying from 0.1 Hz to 1 MHz using two terminal cells consisting of a sandwiched electrolyte layer (200 nm) between the top and bottom Pt electrodes. A detail description about device fabrication for EIS analysis is discussed in experimental section. The thickness and surface analysis of as deposited electrolytes was confirmed with atomic force microscopy (AFM), as shown in Fig. S5 of the Supporting Information. Figure 3(E) show the EIS spectra of Li_3_PO_4_ and Li_3_PO_x_Se_x,_ respectively. The obtained EIS spectra of each electrolyte were appropriately fitted using the standard circuit model (Fig. S6). The fitted EIS data with model circuit clearly shows that the ionic conduction in all electrolytes is modeled by two parallel circuit elements (i.e., constant phase element (CPC) and resistance (R_b_)) in the high and medium frequency regions. The CPE is used to analyze a capacitor; R_b_ used to model an ionic resistance and W_0_ analyze the polarization of the electrode electrolyte interface. The EIS spectra of all electrolytes exhibit a semicircle at the high frequency region and a straight line at the low frequency region. The semicircle in the high frequency region is associated with the ionic movement in the electrolyte, whereas the straight line in the low frequency region is associated with the interface between the electrolyte-Pt electrode. The model circuit consists of one additional elements R_0_ represent the contact resistance. The ionic conductivity of electrolytes is calculated by the equation σ = d/(R_ion_.A), where σ (S cm^−1^) is the ionic conductivity; d (cm) is the thickness; R (Ω) is the resistance; and A (cm^2^) is the area of device. The EIS spectra for Li_3_PO_4_ **(**Fig. 3E**)** clearly show that the large semicircle is associated with more substantial ionic resistance. For Li_3_PO_4_ electrolytes, we measured an ionic conductivity of 1.2 × 10^−7^ S/cm. However, it should be noted that the small semicircle EIS spectra of Li_3_PO_x_Se_x_ **(**Fig. 3E**)** indicates low ionic resistance, which results in high ionic conductivity of 2.0 × 10^−6^ S/cm. Ionic conductivity has been reported to increase by substituting chalcogenide anion, and accordingly, we observed a dramatic increase in ionic conductivity in Li_3_PO_x_Se_x_ electrolytes^25^. We have additionally compare the thin film ionic conducvity of our designed electrolyte with previosuly reported electrolyte matrial and presented in Table S4. The activation energy of Li_3_PO_4_ and Li_3_PO_x_Se_x_ electrolytes were determine using Arrhenius plot of the ionic conductivity as shown in **(**Fig. 3F**)**. The activation energies calculated by using the relation σT = A exp. [−E_a_/(kT)], where σ is the ionic conductivity, T is temperature, A is a pre-exponential factor, Ea is the activation energy, and k is the Boltzmann constant. The plots of ln(σ) vs 1000/T were used to extract the value of activation energy by linearly fitting to the experimental data. From Arrhenius method, the activation energies determined to be 0.356 and 0.253 eV for Li_3_PO_4_ and Li_3_PO_x_Se_x_ electrolytes, respectively. The experimental determined activation energy for ion migration are schematically shown in Fig. 3(G,H). In order to reveal the effect of the Se doping on switching property, we further tune structure of Li_3_PO_x_Se_x_ by changing the Se content. The detailed description on Se content dependent switching behavior of Li_3_PO_x_Se_x_ consisted LIST device can be found in Section 5 of Supplementary Information. At low Se content (0.13%) nonlinear switching with high channel conductance reveal the accumulation of Li^+^ ion at electrolyte/channel interface due low ionic conduction through electrolyte **(**Fig. S7**)**. Another remarkable observation is that when Se content is high (0.52%) saturation in channel conductance was observed, indicating the formation of barrier layer by unreacted Se.

**Figure 4:**
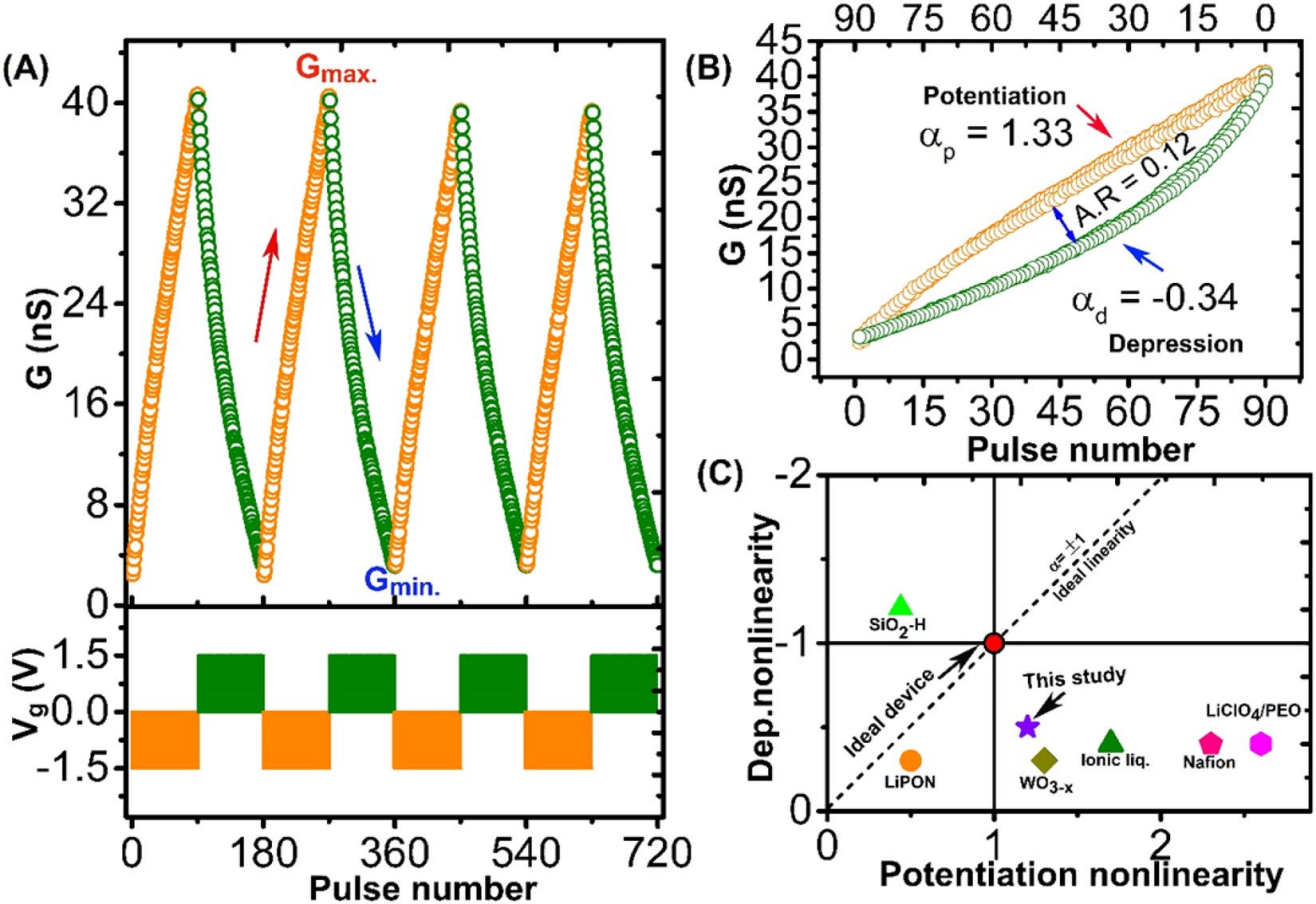
Synaptic functionalities in Li_3_PO_x_Se_x_-based redox transistor. (**A)** Endurance test shows reproducible synaptic weight update (cycling between low and high conductance state) without deterioration in LTP and LTD. Each cycle consists of 90 identical voltage pulses (±1.5 V, 1 s) with 1 s space. **(B)** Analysis of AR between two adjacent LTP and LTD cycles. As a result of the symmetric weight update, a small asymmetric ratio of 0.12 is obtained. Additionally, the device exhibits near ideal linearity for the potentiation and depression steps (α = 1.33/−0.34) (**C)** Comparison of linearity between Li_3_PO_x_Se_x_ electrolyte-based synaptic transistor and previously reported synaptic transistor^1,2,28–31^.

In neuromorphic computing, the endurance of neural hardware is defined using long-term potentiation (LTP) and long-term depression (LTD), which are essential for stable neuromorphic computations. As shown in Fig. 4A, the endurance in Li_3_PO_x_Se_x_-based redox transistor was studied by triggering the LTP and LTD functionalities using an alternate set of 90 identical voltage pulses (±1.5 V, 1 s) with 1 s space. The detail explanation about pulse scheme used for both potentiation and depression are schematically explained in Fig. S8. During the endurance test, channel conductance was reversibly switched between 2.1 to 40.6 nS with the number states representing the good LTP and LTD behaviors, contributing to the high on/off ratio of 19. Moreover, we observed that the device shows negligible degradation of up to 720 write-read cycles, which contribute to good stability of the device. The tendency to preserve the linearity and symmetric weight update during LTP and LTD in a synapse device decides its learning accuracy. To confirm the linearity and symmetric weight update in Li_3_PO_x_Se_x_-based redox transistor, we condensed the LTP and LTD together, as shown in Fig. 4B.

The symmetric weight update between the two consecutive potentiation and depression in a synaptic device is defined by the asymmetric ratio as

$$AR=[\frac{max|{G}_{p}|(n)-max|{G}_{d}|(n)}{{G}_{p(40)}-{G}_{d(40)}}]\,for\,n=1\,to\,40$$

For an ideal device, the asymmetric ratio is zero. The analyzed asymmetric ratio for Li_3_PO_x_Se_x_-based redox transistor is 0.15, which is considerably lower than that of the recently reposted synaptic transistors **(**Table 1**)**. The nonlinearity characteristics shows direct impact on pattern  recognition accuracy when the synaptic device is employed in neuromorphic hardware. Here, nonlinearity calculations are performed using the previously reported weight update formula^26^:

$$\begin{array}{rcl}G & = & \{\,{(({G}_{LRS}^{\propto }-{G}_{HRS}^{\propto })\times W+{G}_{HRS}^{\propto })}^{\frac{1}{\alpha }}\,if\,{\propto }\ne 0\\ G & = & \{{G}_{HRS}\times {(\frac{{G}_{HRS}}{{G}_{HRS}})}^{W}\,if\,{\propto }=\,0\end{array}$$

Where, G_LRS_ and G_HRS_ are the low and high resistance state. The ∝ denote the nonlinearity factor that controls the potentiation or depression. For an ideal device, a perfectly linear and symmetric conductance change occurs, which represents the nonlinearity factor value (∝_p_ = ∝_d_ = 1)^26,27^. The nonlinearity factor values obtained from the fitted LTP and LTD curves were 1.33 and −0.34 for potentiation and depression, respectively. Finally, to compare the performance of Li_3_PO_x_Se_x_-based redox transistor with other synaptic three-terminal transistor devices, we plotted the nonlinearity values obtained from this study and previous studies^1,2,28–31^ **(**Fig. 4C**)**. In particular, this study shows near ideal and symmetric weight update as compared to previously reported ionic transistor, which paves the path of using high ionic conductive electrolyte in future synaptic devices.

**Table 1:** Performance comparison of device level characteristics of several Li^+^ ion-based three-terminal synapse devices with an Li_3_PO_x_Se_x_ device.

Channel material	Electrolyte	Active ion	Linearity of weight update	Conductance^a)^	On/Off ratio	Ref.
WSe_2_	LiClO_4_	Li^+^	0.19	≈570 pS	0.5	^4^
LiCoO_2_	LiPON	Li^+^	0.17	≈250 µS	1.0	^1^
MoO_3_	LiClO_4_	Li^+^	0.31	≈75 nS	1.55	^2^
LiCoO_2_	Li_3_PO_x_Se_x_	Li^+^	0.12	≈40 nS	19	This work

As compared to the previous report on Li^+^ ion-based three-terminal synapse device, Li_3_PO_x_Se_x_-based Li^+^ ion synapse device exhibits excellent synapse characteristics such as lower nonlinearity of weight update  and higher on/off ratio.

^a)^All synaptic deviceshave both low and high conductance level. The given value is for higher conductance level.

**Figure 5:**
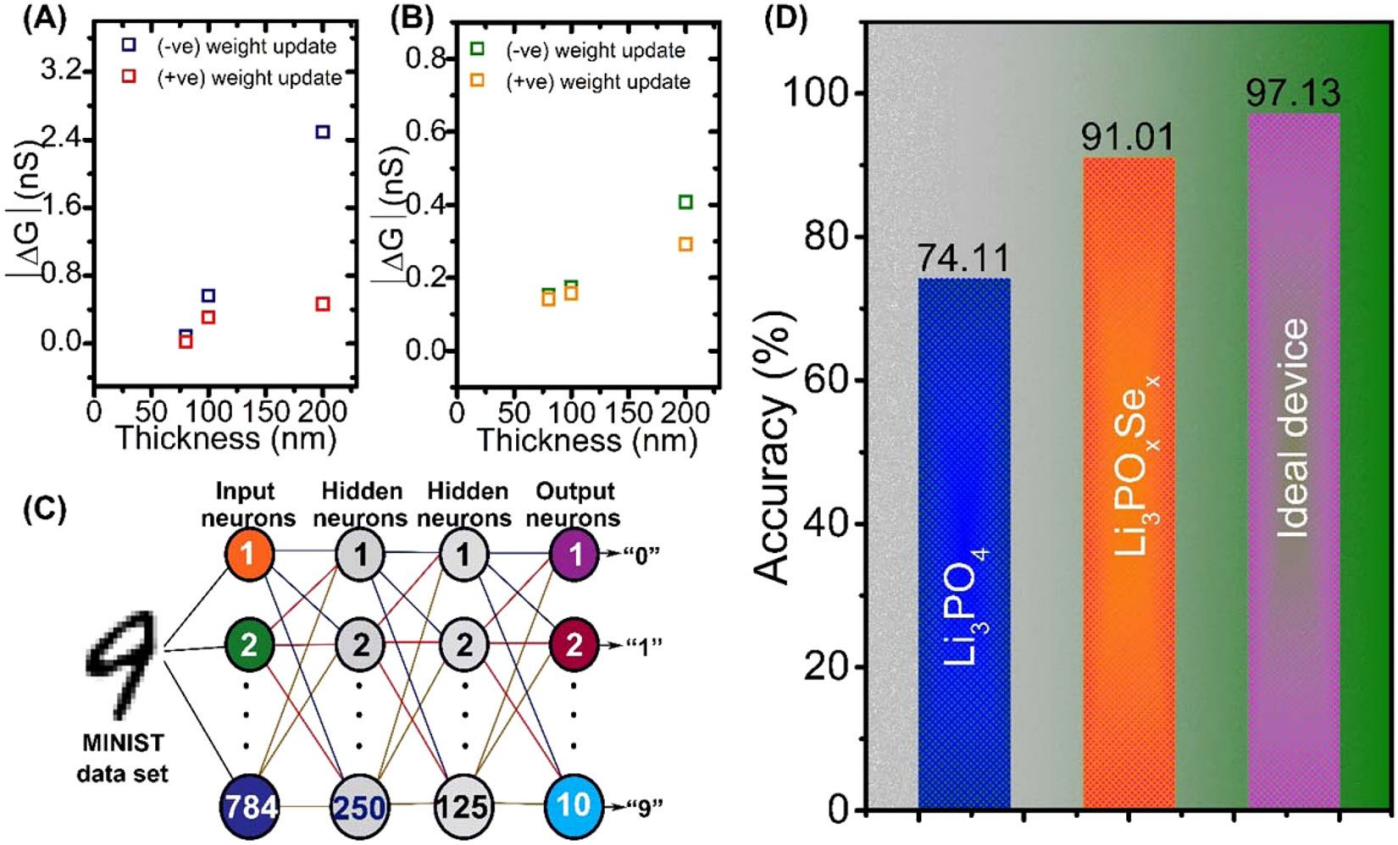
Handwritten image recognition simulation using Li_3_PO_4_ and Li_3_PO_x_Se_x_-based redox transistor parameters. **(A)** The average change in conductance ∆G with the thickness of Li_3_PO_4_ electrolyte varying from 200 to 80 nm. **(B)** The average change in conductance ∆G with the thickness of Li_3_PO_x_Se_x_ electrolyte varying from 200 to 80 nm. **(C)** Schematic of the three-layer artificial neural network, where one layer is hidden and the remaining two layers are for input and output neurons. **(D)** The output result of simulated recognition accuracy of Li_3_PO_x_Se_x_-based redox transistor for large handwritten digit.

Finally, we simulated the artificial neural network of LIST devices using the experimentally determined conductance change value (∆G) and linearity of weight update. To further understand the effect of ∆G on image recognition accuracy, we extracted the average weight change value |∆G| as a function of thickness of electrolytes **(**Fig. 5(A,B)**)**. The effects of linearity and symmetric conductance update of LIST device on the pattern recognition accuracy of a model of a neural network were estimated using multilayer perception based on the Modified National Institute of Standards and Technology dataset.

In the neural network simulation model, the first input, second hidden, and third output layers were composed of 780, 250, 125, and 10 neurons, respectively. All neurons in each layer were fully connected to each other through synapses, as shown in Fig. 5(C) which shows the results of pattern recognition by LIST devices. In the Li_3_PO_x_Se_x_-based LIST device, which exhibits the best synapse performance, the recognition accuracy was significantly improved by 91.01% **(**Fig. 5(D)**)**. The performance comparison between this study and previously reported synaptic transistors is presented in Table 1.

## Conclusion

To summarize, we explored the effect of electrolyte ionic conductivity on the synaptic functionalities in LIST devices. We demonstrated that the substitution of chalcogenide such as Se in oxide-based Li^+^ ion electrolyte enhances the ionic conductivity to 2.0 × 10^−6^ S/cm. The resultant LIST device consisted of high ionically conducting Li_3_PO_x_Se_x_ electrolyte, which exhibits excellent synaptic properties with multiple conductance states at a very low threshold voltage (V_th_ = −2.48 V) and ultralow channel conductance level (40 nA). This LIST device demonstrates symmetric analog switching with a near ideal asymmetric ratio (0.12) and good linearity with nonlinearity 1.33/−0.34 for weight increase /decrease.

## Materials and Methods

### Material synthesis

100 nm of LiCoO_2_ was used as the channel material. It was controllably grown on a 500 nm SiO_2_/Si substrate through reactive sputtering using an LCO target. The nonstoichiometric composition in LiCoO_2_ was controlled by the amount of oxygen during deposition. All LiCoO_2_ samples used in this study were deposited by flowing 1 sccm O_2_ and 30 sccm Ar using a sputtering power of 100 W at 5 mTorr pressure. The solid-state Li_3_PO_4_ and Li_3_PO_x_Se_x_ were used as electrolytes. The electrolyte layers were prepared through reactive sputtering using a 2-inch Li_3_PO_4_ target, whereas Li_3_PO_x_Se_x_ was deposited under Se flux from the Se target. This was followed by the deposition of high-quality 30 nm Si reservoir layer.

### Device fabrication

Initially, various dimensions of source-drain windows were defined on the SiO_2_/Si substrate through photolithography, followed by the deposition of 60 nm pure Pt metal through reactive sputtering and the lift-up process. Then, various dimensions of channel windows were defined on the source-drain pad through photolithography. A 100 nm nonstoichiometric Li_1-x_CoO_2_ channel layer was deposited on the predefined channel windows through reactive sputtering using the LCO target. After the deposition of LiCoO_2_, the lift-up process was performed using acetone to form a LiCoO_2_ channel. Post liftoff, a second lithography step were performed to define the gate electrolyte windows. In the final step, Li_3_PO_4_ or Li_3_PO_x_Se_x_ electrolyte, Si reservoir and Pt metal was deposited on predefined gate electrolyte windows followed by liftoff process.

### Device fabrication for EIS

For EIS measurement Pt/Li_3_PO_4_/Pt and Pt/Li_3_PO_x_Se_x_/Pt sandwiched-cell structure was used. The 100 nm Pt metal deposited on glass substrate as bottom contact electrode. Subsequently, fixed area shadow mask were placed on redeposited bottom electrode and thereafter Li_3_PO_4_ and Li_3_PO_x_Se_x_ electrolytes with ~200 nm thickness were deposited by magneto sputtering. Finally, the 100 nm Pt metal is deposited as top contact electrode. The active device area used in this study is 0.20 cm^2^. The deposited thickness of each electrolyte were controlled by deposition time and it is confirmed by surface analysis technique such as AFM measurement. The result of surface analysis by AFM are shown in Fig. S5 of the Supporting Information.

### Electrical measurement

The measurement of synaptic characteristics was performed in a three-point probe station using a semiconductor parameter analyzer (Agilent B1500) with a waveform generator/fast measurement unit (WGFMU). B1530A WGFMU was used to apply the program voltage pulses for the read and write operations. The conductance of the channel was read using a programmed read pulse using a small DC voltage of 0.5 V between each programming operation to avoid voltage drop in the gate electrolyte.

## Supplementary information

Supplementary information
