# Supplementary material for: Near ideal synaptic functionalities in Li ion synaptic transistor using Li3POxSex electrolyte with high ionic conductivity
Source: Sci Rep. 2019 Dec 11;9:18883. doi: 10.1038/s41598-019-55310-8 (PMC6906484; doi:10.1038/s41598-019-55310-8)
Supplement: Supplementary file 1 — Supplementary information [file 41598_2019_55310_MOESM1_ESM.docx]

**Near ideal synaptic functionalities in Li ion synaptic transistor using Li_3_PO_x_Se_x_ electrolyte with high ionic conductivity**

Revannath Dnyandeo Nikam^1,2^, Myonghoon Kwak^1,2^, Jongwon Lee^1,2^, Krishn Gopal Rajput^1,2^,

Writam Banerjee, Hyunsang Hwang^1,2,*^

^1^Center for Single Atom-based Semiconductor Device, ^2^Department of Material Science and Engineering, Pohang University of Science and Technology (POSTECH), Pohang 790-784, Republic of Korea

** Authors to whom correspondence should be addressed*

*Email:* [*hwanghs@postech.ac.kr*](mailto:hwanghs@postech.ac.kr)

**Section S1: Simulation for time-dependent calculation of Li ion distribution**

To know the amount of Li^+^ transported from LiCoO_2_ channel to Si reservoir via electrolyte (Li_3_PO_4_ OR Li_3_PO_x_Se_x_), we have constructed the one dimensional (1D) simulation model. The model geometry used in this simulation is as shown below.


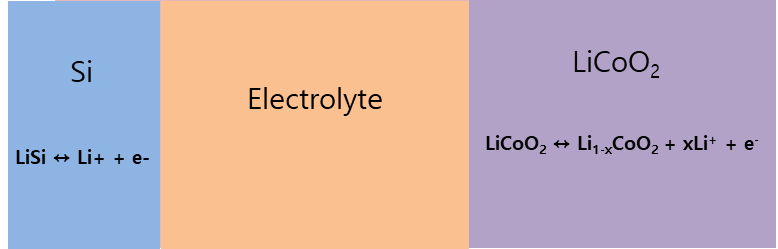


The model geometry consisted 3 domains (channel, electrolyte and Si reservoir).

On the channel side under the electric field the LiCoO_2_ is dissociating as,

LiCoO_2_ ↔ Li_1-x_CoO_2_ + xLi^+^ + e^-^

On the Si reservoir side Li^+^ associate as,

Si + Li^+^ ↔ SiLi

The hysteresis behavior (volatility or nonvolatility) in lithium ion transistor is determined by the dissociation reaction rate in LiCoO_2_ (*k_LCO_*), association rate in Si reservoir (*k_Si_*) and ionic mobility (*µ_ion_*) in electrolyte defined by ionic conductivity of electrolyte.

The reaction kinetics in LiCoO_2_ channel and in Si reservoir are described using the Butler-Volmer equation as,

*i_0_*_, LiCoO2_ =Fk_LiCoO2_ $\left( \frac{CLi,max-CLi)CLi}{CLi,max-CLi,min)CLi} \right)^{LiCoO2} \left( \frac{CLi,-CLi,min)}{CLi,max-CLi,min)} \right)^{1-LiCoO2}$

Where C_Li,max_ and C_Li,min_ are the maximum and minimum amount of Li^+^ ion in LiCoO_2_ channel.

And --------------(1)

*i_0_*_, Si_ =Fk_Si_ $\left( \frac{CLi,max-CLi)CLi}{CLi,max-CLi,min)CLi} \right)^{\mathrm{Si}} \left( \frac{CLi,-CLi,min)}{CLi,max-CLi,min)} \right)^{1-\mathrm{Si}}$

During the potentiation, a negative gate pulses initiate the oxidation reaction at the surface of LiCoO_2_ and produces the Li^+^ ions. Under the electric field this Li^+^ migrate toward Si reservoir via electrolyte.

The chemical reaction in electrolyte is as,

Li^+^ ↔ Li^0^ + n^-^

The transport Li^+^ kinetic in electrolyte are described by the Nernst-Plank equation as,

N_i =_ -D_i_ 𝐷𝑖∇𝑐 +$\frac{ZiF}{RT}$ DiCi ∇𝜙_l_ ----------------(2)

The hysteresis behavior (volatility or nonvolatility) of Li_3_PO_4_ and Li_3_PO_x_Se_x_ electrolytes consisted synaptic transistor devices can have explained by knowing the Li^+^ ion concertation with time in LiCoO_2_ after the potentiation. To know the Li^+^ ion concertation with time in LiCoO_2_, we solve the Equation (1) and (2) using COMSOL multiphysic simulation platform with experimentally know parameters such as thickness of LiCoO_2_ channel, electrolyte and Si reservoir.

We first experimentally obtained the voltage decay curve with time for device with constant current. Then we simulate the device by tuning the various parameter to know which parameters were the most important in governing the time decay behavior. We finally concluded that the diffusion rate and reaction rates are the important parameter. Then we tuned the parameters during simulation until we obtain better fit to our experimental result.





**Figure S1:** Experimental and simulation voltage decay curves for Li_3_PO_4_ and Li_3_PO_x_Se_x_ electrolytes.





**Figure S2:** Li^+^ ion concentration in LiCoO_2_ after removal of current bias in Li_3_PO_4_ and Li_3_PO_x_Se_x_ consisted devices.

**Table S1:** The parameter used in simulation is given in below table.

| L | Thickness of electrolyte | 200 [nm] |
| --- | --- | --- |
| M | Thickness of electrode | 100 [nm] |
| c0_Li_ion | Total concentration of Li ions in Li_3_PO_4_ or Li_3_PO_x_Se_x_ | 6.01×10^4^ [mol/mm^3^] |
| kr | Li ion recombination reaction rate | 0.9×10^-8^ -[m^3/(mol*s)] |
| delta | Fraction of free Li ions in equilibrium | 0.18 |
| D_Li_ion | Diffusion coefficient for Li ions in electrolyte | 0.9e-15[m^2/s] |
| D_n | Diffusion coefficient for n in electrolyte | 5.1e-15[m^2/s] |
| c_Li_max | Selected maximal activity of Li, positive electrode | 2.33e4[mol/m^3] |
| D_Li | Diffusion coefficient for Li, positive electrode | 1.76e-15[m^2/s] |
| alpha_pos | Charge transfer coefficient | 0.6 |
| k_LCO | Rate constant charge transfer reaction, positive electrode | 5.1e-4[mol/m^2/s] |
| T | Temperature | 298.15[K] |
| kd | Dissociation rate constant in electrolyte | kr*c0_Li_ion*delta^2/(1-delta) |
| c_Li_ion_init | Initial Li ion electrolyte concentration | c0_Li_ion*delta |
| i_1C | 1C current | 10e-6[A/cm^2] |
| c_Li_init | Initial Li concentration, positive electrode | c_Li_max/2*1.01 |
| C_rate | C rate parameter in parametric sweep | 1 |
| c_Li_min | Minimum Li concentration, positive electrode | c_Li_max/2 |
| alpha_neg | Charge transfer coefficient, negative electrode | 0.5 |
| k_neg | Rate constant charge transfer reaction, negative electrode | 1e-2[mol/m^2/s] |

**Section S3: XPS analysis of Li_3_PO_4_ and Li_3_PO_x_Se_x_**





**Figure S3**. Survey XPS spectra of Li_3_PO_4_ deposited at 30^0^C.





**Figure S4**. Survey XPS spectra of Li_3_PO_x_Se_x_ deposited at 30^0^C.

**Table S2.** Fractional % elemental analysis in Li_3_PO_4_ electrolyte from the XPS spectra

| **Element** | **B.E.** | **Height CPS** | **FWHM** | **Area CPS** | **Atomic %** | **Structure** |
| --- | --- | --- | --- | --- | --- | --- |
| Li (1s) | 56.48 | 1969.19 | 1.97 | 4532.53 | 53.06 | Li_3_PO_4_ |
| C (1S) | 290.83 | 3498.82 | 1.37 | 7218.24 | 5.05 |  |
| P (2p) | 134.31 | 5061.2 | 1.91 | 10262.37 | 5.83 |  |
| O (1s) | 532.41 | 68024.47 | 1.78 | 141568.23 | 36.06 |  |

# Fractional % element calculated from % = (the peak area under (XPS elemental peak)/the sensitivity factor of element ) × 100.

**Table S3.** Fractional % elemental analysis in Li_3_PO_x_Se_x_ electrolyte from the XPS spectra

| **Element** | **B.E.** | **Height CPS** | **FWHM** | **Area CPS** | **Atomic %** | **Structure** |
| --- | --- | --- | --- | --- | --- | --- |
| Li1s | 56.22 | 4143.09 | 1.71 | 7725.61 | 53.74 | Li_3_PO_x_Se_x_ |
| P2p | 134.11 | 1476.59 | 1.77 | 2835.43 | 0.96 |  |
| C1s | 291 | 16956.21 | 1.24 | 27941.82 | 11.61 |  |
| Se3p | 161.87 | 1112.28 | 2.37 | 2957.83 | 0.26 |  |
| O1s | 532.81 | 125313.05 | 1.59 | 220876.01 | 33.44 |  |

# Fractional % element calculated from % = (the peak area under (XPS elemental peak)/the sensitivity factor of element) × 100

**Section S5: Surface and thickness analysis of Li_3_PO_4_ and Li_3_PO_x_Se_x_ using AFM**


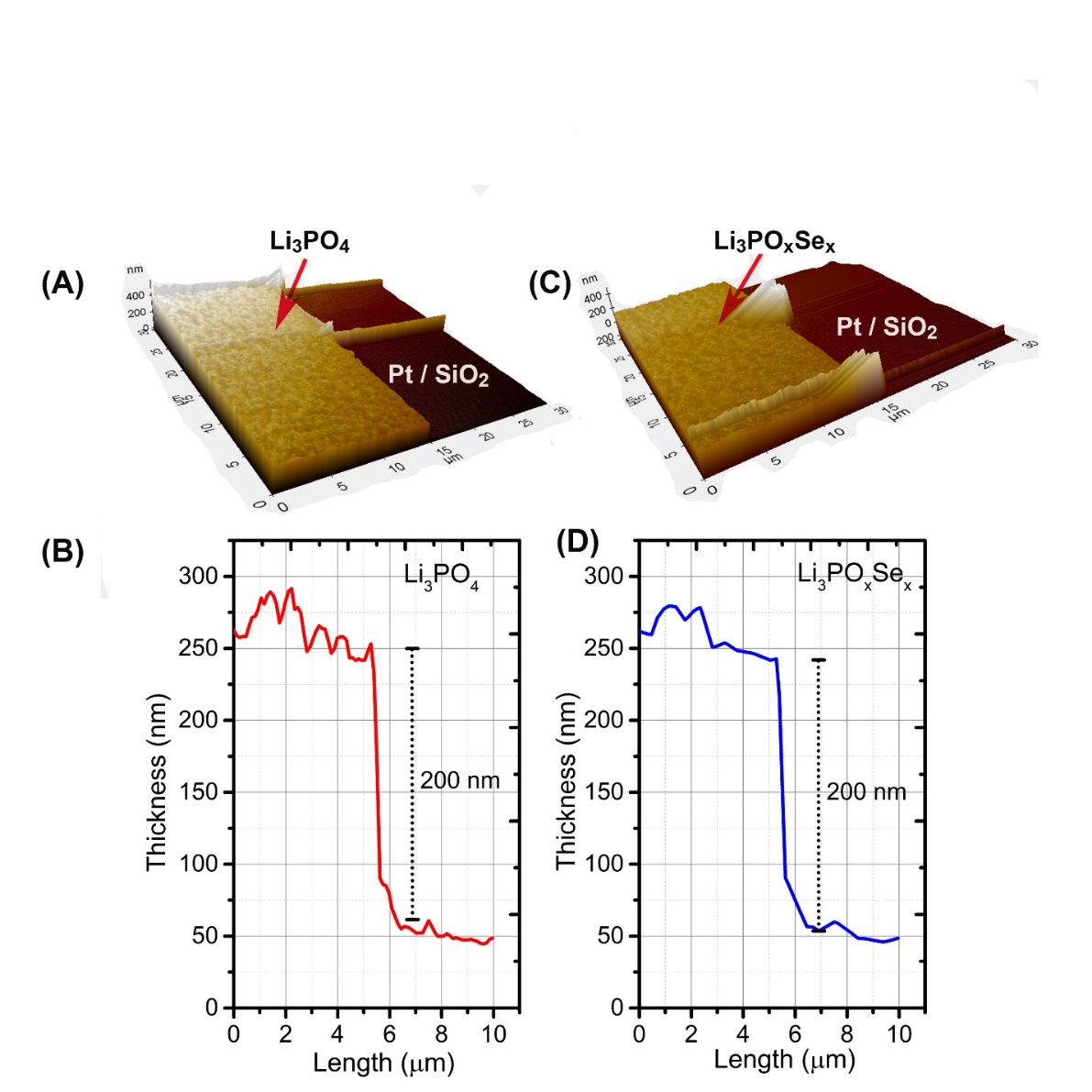


**Figure S5:** **(A-B)** Surface analysis of as grown electrolytes film by atomic force microscopy (AFM). **(B-D)** Depth profile to demine the exact thickness of as deposited electrolyte.

**Section S6: EIS circuit model**


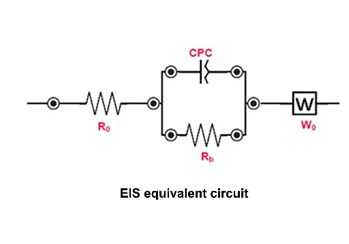


**Figure S6:** EIS circuit model used to fit the raw data.

**Section S7: Se content dependnt swtching behavior of Li_3_PO_x_Se_x_ consisted LIST device.**





**Figure S7:** LTP and LTD characteristics Li_3_PO_x_Se_x_ with varying Se content

The obtained synaptic property with varying Se content in Li_3_PO_x_Se_x_ is summarized in below table.

| % Se content | Initial conductance (nS) | On/off ratio | Asymmetric ratio |
| --- | --- | --- | --- |
| 0.13 | 2.7 | 11 | 1.5 |
| 0.26 | 2.6 | 13 | 0.15 |
| 0.52 | 4.1 | 1.8 | 0.95 |

Note: In ideal symmetric switching the asymmetric ratio is zero.

To change the Se content Li_3_PO_x_Se_x_ electrolyte, we have change the RF power of Se target during deposition. To correlate the RF power and Se content in Li_3_PO_x_Se_x_ we use high resolution XPS analysis. First we fabricated a reference sample of Li_3_PO_x_Se_x_ by giving 10 W RF power to Se target and analyzed with XPS. The obtained XPS result on Li_3_PO_x_Se_x_ reference samples is as below.

**Section S8: Individual pulse scheme for endurance test**





**Figure S8**. Schematic of individual pulse scheme used for potentiation and depression during endurance test. During each potentiation and depression cycle 90 pulses with (±1.5 V, 1 s) with 1 s space were used.

**Table S4:** Comaparision of ionic conductiviyt of various electrolytes with present study.

| **Solid-State electrolyte** | **Thickness** | **Li+ conductivity (S cm−1)** | **Composition** | **Method** | **References** |
| --- | --- | --- | --- | --- | --- |
| Li_3_PO_4_ | 1000 nm | 7 × 10−8 (25°C) | Li_2.7_PO_3.9_ | RF sputtering | [1] |
| Li_3_PO_4_ | 130 ± 10 nm | 3.3 × 10−8 (25°C) | Li_2.8_PO_2_ | ALD | [[2](#_bookmark43)] |
| Li_3_PO_4_ | 1000 nm | 6.6 × 10−8 (25°C) | Li_2.7_PO_3.9_ | RF sputtering | [3] |
| Li_3_PO_4_ | 2000 nm | 4 × 10−7 (25°C) | Li_3_PO_4_ | PLD | [[4](#_bookmark44)] |
| Li_3_PO_4_ | 1500 nm | 3.3 × 10−7 (25°C) | Li_3_PO_4_ | PLD | [[5](#_bookmark44)] |
| Li_3_PO_4_  (polycrystalline) | 0.2 cm | 4.2 × 10−18 (25°C) | Li_3_PO_4_ | Solid state reaction | [[6](#_bookmark44)] |
| Li_3_PO_4_  (N_2_ doping) | 2000 nm | 2.1 × 10−6 (25°C) | Li_2.9_ PO_2.6_ N_0.91_ | RF sputtering | [[7](#_bookmark44)] |
| Li_3_PO_4_  (N_2_ doping) | 2000 nm | 1.7 × 10−6 (25°C) | Li_1.8_ PO_1.2_ N_1.5_ | RF sputtering | [[7](#_bookmark44)] |
| Li_3_PO_4_  (N_2_ doping) | 2000 nm | 3.1 × 10−6 (25°C) | Li_3.3_ PO_2.9_ N_0.83_ | RF sputtering | [[7](#_bookmark44)] |
| Li_3_PO_4_  (N_2_ doping) | 12 nm | 2.71 × 10−10 (25°C) | 0% N content | ALD | [[8](#_bookmark44)] |
| Li_3_PO_4_  (N_2_ doping) | 10 nm | 3.36 × 10−7 (25°C) | 10 % N content | ALD | [[8](#_bookmark44)] |
| Li_3_PO_4_  (Si doping) | 1000 nm | 1.49 × 10−6 (25°C) | Li_2.40_ Si_0.88_P_0.12_O_2.28_ | RF sputtering | [[9](#_bookmark44)] |
| Li_3_PO_4_  (Si and N_2_ doping) | 1000 nm | 2.06 × 10−5 (25°C) | Li_1.35_ Si_0.79_P_0.21_O_1.98_N_0.98_ | RF sputtering | [[9](#_bookmark44)] |
| Li_3_PO_4_ | 200 nm | 1.2 × 10^-7^ (25°C) |  | RF sputtering | This study |
| Li_3_PO_4_  (Se doping) | 200 nm | 2.0 × 10^-6^ (25°C) |  | RF sputtering | This study |

*[****1****] J. Electrochem. Soc., 144 (1997), pp. 524-532; [****2****] Nanotechnology 25 (2014) 504007; [****3****] J. Power Sources 44 103–10; [****4****] Journal of The Electrochemical Society, 157 (4) A521-A527 (2010); [****5****] ECS Transactions, 16 (26) 53-60 (2009); [****6****] J. Solid State Chem. 115 313–23; [****7****] Solid State Ionics 191 (2011) 49–54; [****8****] Chemistry of Materials 2015 27 (15), 5324-5331; [****9****] ACS Appl. Energy Mater. 2019, 2, 4782−4791*
